# The Effect of Physical Activity on Postural Abilities in Menopausal Women

**DOI:** 10.1192/j.eurpsy.2024.1485

**Published:** 2024-08-27

**Authors:** A. Feki, I. Sellami, F. Ellouze, M. Yahya, S. Baklouti, M. H. Elleuch

**Affiliations:** ^1^Rheumatology; ^2^Occupational medicine, Hedi Chaker Hospital; ^3^Physical Medicine and Functional Rehabilitation, Habib Bourguiba Hospital, Sfax, Tunisia

## Abstract

**Introduction:**

Menopause marks a significant physiological transition in a woman’s life, often accompanied by various health challenges. Understanding the impact of physical activity on postural abilities in menopausal women is crucial for promoting their overall well-being during this transformative stage.

**Objectives:**

The aim of our study was to investigate the effect of a physical activity program on postural abilities, psychological well-being, and the quality of life of menopausal women.

**Methods:**

Nineteen menopausal women, averaging 56±3 years of age, participated in a 12-week Zumba-style physical training program, consisting of three 50-minute sessions per week. The exercise regimen incorporated aerobic workouts, muscle conditioning, balance exercises, and flexibility training, predominantly inspired by Latin dances. Postural balance was evaluated using a stabilometric force platform, measuring the average velocity of the center of pressure (COPvm) under open eyes (OE) and closed eyes (CE) conditions on both firm and soft surfaces. Quality of life and mood were assessed using the SF-36 questionnaire (Short Form Survey-36) and the BMIS score (Brief Mood Introspection Scale). Assessments were conducted before (pre-test) and after (post-test) the 12-week training period.

**Results:**

The findings revealed a significant decrease in COPvm values in the post-test for both conditions (on a firm surface: OE p=0.05, CE p=0.01; on a soft surface: OE p=0.001, CE p=0.05). Additionally, improvements in mood (p=0.05) and quality of life (p=0.05) were observed compared to baseline values.

**Conclusions:**

This study underscores the positive impact of Zumba-style physical training on postural abilities, mood, and quality of life among menopausal women. These results suggest that such exercise programs hold promise in reducing the risk and incidence of falls associated with menopause

**Disclosure of Interest:**

None Declared

